# Application of Intracranial Pressure-Directed Therapy on Delayed Cerebral Ischemia After Aneurysmal Subarachnoid Hemorrhage

**DOI:** 10.3389/fnagi.2022.831994

**Published:** 2022-03-14

**Authors:** Jun Yang, Junlin Lu, Runting Li, Fa Lin, Yu Chen, Heze Han, Debin Yan, Ruinan Li, Zhipeng Li, Haibin Zhang, Kexin Yuan, Hongliang Li, Linlin Zhang, Guangzhi Shi, Jianxin Zhou, Shuo Wang, Yuanli Zhao, Xiaolin Chen

**Affiliations:** ^1^Department of Neurosurgery, Beijing Tiantan Hospital, Capital Medical University, Beijing, China; ^2^Department of Critical Care Medicine, Beijing Tiantan Hospital, Capital Medical University, Beijing, China; ^3^China National Clinical Research Center for Neurological Diseases, Beijing, China; ^4^Center of Stroke, Beijing Institute for Brain Disorders, Beijing, China; ^5^Beijing Key Laboratory of Translational Medicine for Cerebrovascular Disease, Beijing, China; ^6^Beijing Translational Engineering Center for 3D Printer in Clinical Neuroscience, Beijing, China

**Keywords:** aneurysmal subarachnoid hemorrhage, intracranial pressure, delayed cerebral ischemia, delayed neurological deterioration, dehydration

## Abstract

**Objective:**

Elevated ICP is a well-recognized phenomenon in aneurysmal subarachnoid hemorrhage (aSAH) that has been demonstrated to lead to poor outcomes. Delayed cerebral ischemia (DCI) is the most important reason for a poor clinical outcome after a subarachnoid hemorrhage. DCI is understood as a multifactorial process that evolves over time, largely caused by the sequelae of increased intracranial pressure (ICP). The study aimed to assess how to better define the association between ICP and DCI, and whether rational ICP management can improve the outcome of aSAH patients.

**Methods:**

We prospectively reviewed patients diagnosed with aSAH and received microsurgery clipping at our department from December 2019 to January 2021. Subdural ICP monitoring devices were kept to monitor the ICP. The ICP values were recorded every 1-h epochs. ICP -guided dehydration treatments were routinely performed to control the ICP level of patients after surgery. To evaluate whether the subdural ICP-directed management improved the prognosis of the aSAH patients, we compared the outcome data of the patients in our cohort with those treated at another ward of our hospital at the same period.

**Results:**

In total, 144 consecutive aSAH patients received microsurgery clipping at our department, 68 of whom underwent ICP monitoring. A total of 11,424 1-h ICP measurements were recorded for the included patients (1.30 years of recordings). Of 68 patients with ICP monitoring, 27 (27/68, 39.7%) patients developed DCI. Univariate analysis showed that higher Hunt-Hess grade (OR 2.138, 95% CI 1.025–4.459, *p* = 0.043), higher preoperative modified Rankin Scale score (OR 1.534, 95% CI 1.033–2.276, *p* = 0.034), and the max ICP value of each day value >28.5 mmHg (OR 4.442, 95% CI 1.509–13.082, *p* = 0.007) were associated with DCI. Also, patients with ICP-directed treatment showed a significantly lower DCI incidence than patients without ICP monitoring.

**Conclusion:**

Our study suggests that I less than 15 mmHg possibly constitute normal values and that 28.5 mmHg is the ICP threshold most strongly associated with the occurrence of DCI in aSAH patients. Patients who received the ICP-directed treatment presented a lower incidence of DCI. Our findings provide a basis for the recommendation of ICP-directed treatment after aSAH.

**Trial Registration Number:**

NCT04785976.

## Introduction

Aneurysmal subarachnoid hemorrhage (aSAH) constitutes a life-threatening subtype of stroke affecting patients at a mean age of 55 years, leading to loss of many years of productive life, which account for more than 85% of all subarachnoid hemorrhage without preceding trauma (Macdonald and Schweizer, 2017). Better diagnosis, early aneurysm repair, prescription of nimodipine, and advanced intensive care support increased the survival from aSAH in the past few decades. Nevertheless, patients with aSAH still have a high disability rate, affecting patients’ daily functionality, working capacity, and quality of life (Florez et al., 2021). The neurological outcome for aSAH patients is seriously influenced by the development of delayed cerebral ischemia (DCI) (Stienen et al., 2018), but adequate treatments to prevent DCI remain elusive. Currently, DCI is understood as a multifactorial process that evolves over time, largely caused by the sequelae of increased intracranial pressure (ICP) and transient global ischemia during ictus (Dodd et al., 2021). Elevated ICP is a well-recognized phenomenon in aSAH that has been demonstrated to lead to poor outcomes (Fukuhara et al., 1998; Wang et al., 2014). However, there are no consensus guidelines devoted specifically to the management of elevated ICP in the setting of aSAH. Most centers extrapolate their treatment algorithms from studies and published guidelines for traumatic brain injuries to treat ICP elevation in aSAH (Alotaibi et al., 2017). The threshold at which ICP begins to exhibit detrimental effects is not known with certainty. Likewise, it remains unclear what the ideal treatment threshold for ICP is and whether a common threshold should be used for all patients and pathologies. There is a lack of recommendations regarding the indications for ICP monitoring in patients with aSAH. Thus, using our prospective database of high-frequency physiologic data, our group has endeavored to better define the association between ICP and DCI, and whether rational ICP management can improve the outcome of aSAH patients.

## Materials and Methods

### Study Design

The study was approved by the Beijing Tiantan Hospital Research Ethics Committee. Informed consent was obtained from the patient or surrogate decision-maker before enrollment. We prospectively reviewed patients diagnosed with aSAH and received microsurgical clipping at our department from December 2019 to January 2021.

We routinely transferred the patients to the intensive care units after the aneurysms were treated with microsurgical clipping. Subdural ICP monitoring devices (MicroSensor Basic Kit, Codman, United States) were kept to monitor the ICP. The ICP values were recorded every 1-h epochs. The max ICP value of each day was defined as ICPmax. The treatments of ICP elevation were performed when ICP values exceeded 20 mmHg for more than 1 h, including raising the patients’ head, sedation, hyperventilation, and dehydration [intravenous drip of mannitol (0.25 g/kg, Q6H)]. The duration of ICP monitoring also varied widely, patients with worse neurological function had longer monitoring periods, but it was at least more than 7 days. Generally, ICP probes were removed after 24 h of ICP less than 20 mmHg without any dehydration treatments. If the neurological function of patients deteriorated, CT scans or angiography were performed to detect the occurrence of delayed cerebral ischemia (DCI). Otherwise, a CT scan was performed to evaluate whether new silent infarcts before the patients left the intensive care unit (ICU).

In addition, ICP-directed therapy was not routinely performed in all aSAH patients after surgery in our institution. Another ward in our institution for surgical treatment of aSAH rarely uses ICP monitoring after surgery, but other postoperative medical treatment aside from the ICP monitoring and associated therapies didn’t differ between the two wards. Thus, to further evaluate whether the subdural ICP monitoring devices guided ICP management improved the prognosis of the aSAH patients, we compared the outcome data of the patients in our cohort with those consecutively treated at another ward of our hospital at the same period.

### Inclusion and Exclusion Criteria

The inclusion criteria included: (1) patients who were diagnosed with aSAH by computed tomography (CT) and digital subtraction angiography or computed tomography angiography (CTA), (2) adult patients (>18 years of age), (3) the aneurysms were treated by microsurgery clipping and subdural ICP monitoring probes performed, (4) subarachnoid hemorrhage diagnosed to treated by microsurgery less than 72 h. (5) Hunt-Hess grade 1–4. Patients combined with congenital cerebral vascular disease (e.g., arteriovenous malformations and moyamoya disease) and with coexistent intracranial lesions were simultaneously treated (e.g., resection of meningioma or pituitary adenoma) were excluded from the present study.

### Procedures

All patients underwent CTA on admission. A plain CT scan was performed for each patient to check intracranial conditions after surgery. Additional CT scans would be performed when patients had new symptoms of deteriorating neurological function. Imaging data were collected from the imaging system. The size of the aneurysm was measured as its maximal diameter on CTA images, and the location of the aneurysm was grouped according to its parent artery.

Post-operative CTA was conducted 7–10 days before discharge when patients could tolerate the examination. Demographics and Clinical information were collected from the electronic medical record system, including age, sex, history of hypertension, diabetes, hyperlipidemia, coronary disease, past ischemic and hemorrhagic stroke, and smoking and drinking status.

### Outcome Assessment

Baseline clinical characteristics and imaging data were reviewed, including age, sex, location of the ruptured aneurysm, and medical and medication history. The Hunt-Hess (H-H) grade, modified Fisher (mFS) grade, and modified Rankin Scale (mRS) score were assessed. Postoperative clinical complications during hospitalization were collected, including intracerebral hemorrhage, postoperative infarction, infection, DCI, hydrocephalus. We used the mRS score to measure the level of neurological function. For the patients with an mRS score ranging from 0 to 2 on admission, disability was defined as a final score of 3, 4, or 5 (moderate to severe); for patients with preoperative mRS score higher than 2, disability was described as an increase of 1 or more scores.

### Statistical Analysis

All statistical analyses were performed using SPSS Statistics 26.0 (IBM, Armonk, NY, United States) and GraphPad PRISM 8.3.0 (GraphPad Software Inc., San Diego, CA, United States). Statistical significance was set at *p* < 0.05 for 95% CI. The descriptive statistics were summarized as mean ± standard deviation for continuous variables. After testing for normality, continuous variables were analyzed using the independent Student *t*-test (normal distribution) or Mann–Whitney *U* test. The Pearson chi-square test, continuity correction test or Fisher’s exact test were used to test the dichotomized and categorical independent variables. A multivariate logistic regression analysis was conducted to test the effects of various parameters on DCI occurrence. The area under the receiver operating characteristic curve (ROC) was calculated to measure each independent predictor’s prediction ability.

Propensity score matching (PSM) was carried out to adjust for potential baseline confounding characteristics when comparing postoperative complications and discharge outcomes between the ICP and non-ICP monitoring groups, including age, sex, location of the ruptured aneurysm, and HH grade. Using the nearest-neighbor method without replacement for propensity score matching, pairs of patients were matched with a match tolerance of 0.02 and a ratio of 1:1. The postoperative complications and discharge outcomes were compared between aSAH patients with and without ICP monitoring in the match pairs.

## Results

### Association Between Intracranial Pressure and Delayed Cerebral Ischemia

We recorded data of 144 consecutive aSAH patients who received microsurgery clipping at our department, 68 of whom underwent ICP monitoring. Postoperative complications and outcome data were available for all patients. A total of 11,424 1-h ICP measurements were recorded for the included patients (1.30 years of recordings). Patient demographics are presented in Table 1.

**TABLE 1:** Baseline characteristics.

Characteristic	Total (68)	DCI (27)	Non-DCI (41)	*p* values
Age, years	53.5 ± 10.5	52.6 ± 11.9	54.2 ± 9.6	0.577
Sex				0.347
Male	33 (48.5)	15 (55.6)	18 (43.9)	
Female	35 (51.5)	12 (44.4)	23 (56.1)	
H-H grade				0.106
1	3 (4.4)	0 (0)	3 (7.3)	
2	49 (72.1)	17 (63.0)	32 (78.1)	
3	9 (13.2)	6 (22.2)	3 (7.3)	
4	7 (10.3)	4 (14.8)	3 (7.3)	
mFS grade				0.548
2	20 (29.4)	6 (22.2)	14 (34.1)	
3	24 (35.3)	10 (37.1)	14 (34.1)	
4	24 (35.3)	11 (40.7)	13 (31.8)	
mRS score				0.036
1	54 (79.4)	17 (63.0)	37 (90.3)	
2	1 (1.5)	1 (3.7)	0 (0)	
3	3 (4.4)	2 (7.4)	1 (2.4)	
4	4 (5.9)	4 (14.8)	0 (0)	
5	6 (8.8)	3 (11.1)	3 (7.3)	
Location				0.156
ICA	5 (7.4)	3 (11.1)	2 (4.9)	
MCA	29 (42.7)	7 (25.9)	22 (53.7)	
ACoA	24 (35.3)	13 (48.2)	11 (26.8)	
PCoA	9 (13.1)	4 (14.8)	5 (12.2)	
ACA	1 (1.5)	0 (0)	1 (2.4)	
Smoking	10 (14.7)	6 (22.2)	4 (9.8)	0.156
Drinking	6 (8.8)	4 (14.8)	2 (4.9)	0.158
Hypertension	32 (47.1)	11 (40.7)	21 (51.2)	0.397
Diabetes	1 (1.5)	0 (0)	1 (2.4)	0.414
Hyperlipidemia	1 (1.5)	1 (3.7)	0 (0)	0.214
ICP_max_, mmHg	25.5 ± 10.1	30.1 ± 12.7	22.4 ± 6.5	0.002

*H-H grade, Hunt-Hess grade; mFS grade, modified Fisher grade, mRS score, modified Rankin Scale score; ICA, internal carotid artery; MCA, middle cerebral artery; ACoA, anterior communicating artery; PCoA, posterior communicating artery; ACA, anterior cerebral artery; ICP, intracranial pressure. Data are n (%) unless otherwise indicated. Mean values are given with SDs.*

**FIGURE 1:**
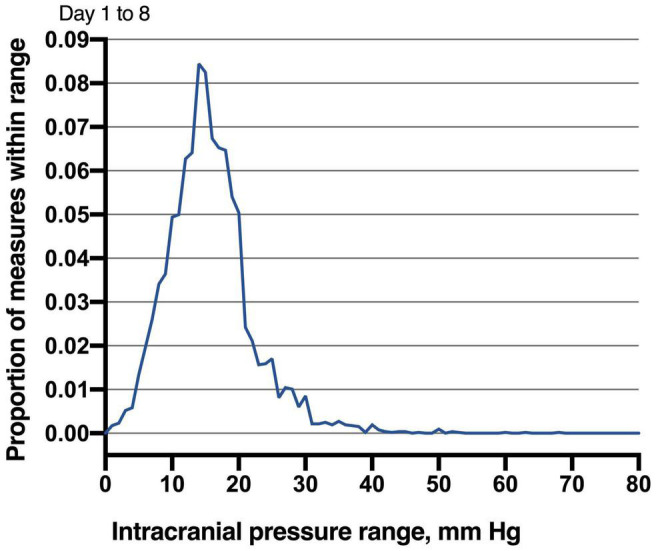
Distribution of intracranial pressure measures in studied patients. Values obtained between days 1 and 8 following microsurgery to the neurocritical care unit are reported. The most common ICP (nearest integer) measured in all patients from day 1 to 8 was 14 mmHg (8.44% of all measures). Given the robust mode demonstrated by these distributions, ICP values less than 15 mmHg may be normal.

**FIGURE 2:**
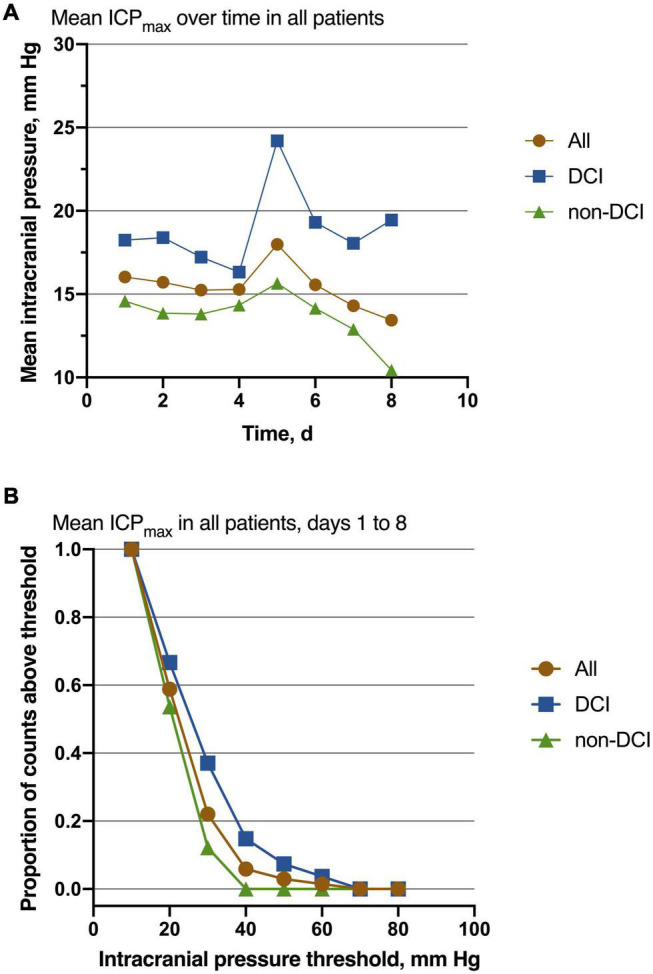
Association of ICP values with the outcome. **(A)** The most common mean ICPmax over time measured in all patients from day 1 to 8 was 18 mmHg, while in patients with DCI it was 24 mmHg, and non-DCI was 16 mmHg. All groups had the peak value at about the 5th day. Patients with DCI had higher mean values than those with non-DCI. **(B)** Mean ICPmax values are shown for decreasing function from the Intracranial pressure threshold increasing, especially at the point of 30 mmHg. For all patients, *n* = 68; DCI = 27, non-DCI = 41.

**FIGURE 3:**
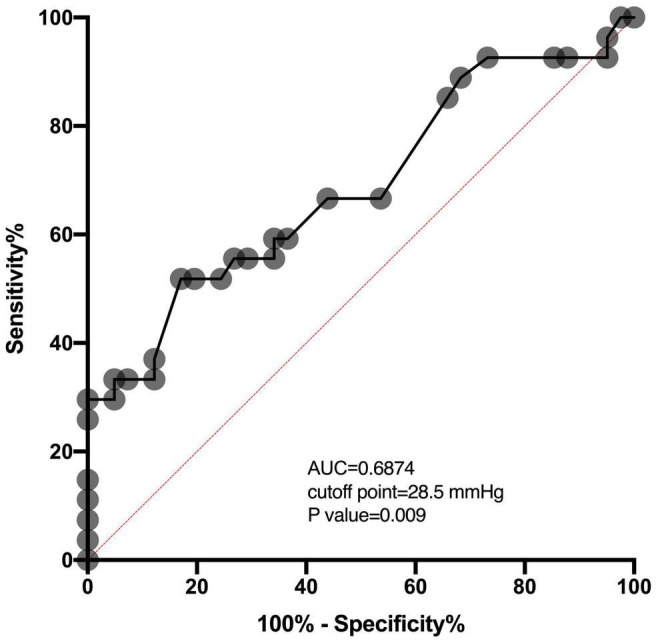
ROC analysis shows the AUC of the ICPmax values for the prediction of DCI. The AUC of the ICPmax values was 0.6874. The best cutoff value was 28.5 mmHg providing sensitivity and specificity of 51.85 and 80.49%, respectively. The red dashed line between location (0,0) and (1,1) is a baseline. AUC above this line means above 0.5 and means a better outcome.

Of 68 patients with ICP monitoring, 27 (27/68, 39.7%) patients developed DCI. Patients with DCI tended to have high mRS scores on admission and higher postoperative ICPmax values. We thoroughly analyzed ICP values in different epochs from DCI patients and non-DCI patients. Salient data for days 1–8 are shown in Figure 1. A discrete peak in ICP values was seen at approximately 14 mmHg, a possible indication of normal ICP. A sharp drop-off in ICP was consistent around the treatment proportion of counts above the threshold of 20 mmHg, reflecting efforts to treat higher values. Nonetheless, 21.1% of all measures were greater than 20 mmHg. Mean ICPmax values were associated with DCI (Figures 2A,B). The most common mean ICPmax over time measured in all patients from day 1 to 8 was 18 mmHg, while in patients with DCI it was 24 mmHg, and non-DCI was 16 mmHg. All groups had the peak value at about the 5th day. Mean ICPmax values were generally higher in patients with DCI than those with other conditions. Moreover, a ROC curve identified that a ICPmax value >28.5 mmHg were associated with DCI in aSAH patients with 51.85% sensitivity and 80.49% specificity [area under curve (AUC), 0.69; 95% CI, 0.660–0.898; *P* = 0.009] (Figure 3).

### Analysis of Potential Delayed Cerebral Ischemia Risk Factors for Aneurysmal Subarachnoid Hemorrhage Patients

**FIGURE 4:**
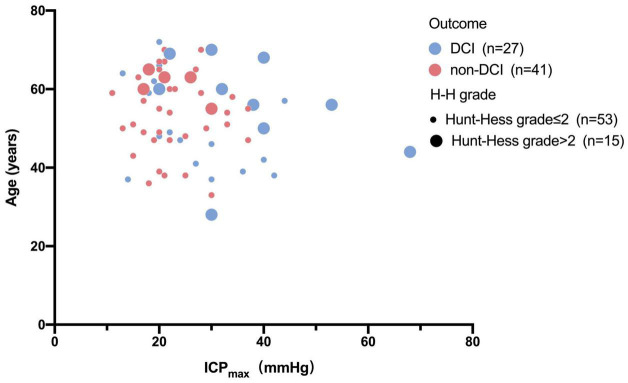
Bubble chart of ICP_max_ values and pre-operation status with the outcome. This Bubble chart shows that patients with higher ICP_max_ values and H-H grade more than two scores and older are more likely to develop DCI than those with lower Hunt-Hess grade and lower ICP_max_ values.

Univariable and multivariable ORs for the risk factors of DCI are shown in Table 2. Univariate analysis showed that higher H-H grade (OR 2.138, 95% CI 1.025–4.459, *p* = 0.043), higher preoperative mRS score (OR 1.534, 95% CI 1.033–2.276, *p* = 0.034), and ICPmax value >28.5 mmHg (OR 4.442, 95% CI 1.509–13.082, *p* = 0.007) were associated with DCI. However, after adjusting for potential covariables, multivariate analysis showed only max ICP value >28.5 (OR, 3.538; 95% CI, 1.140–10.986; *P* = 0.029) was associated with a significantly increased risk of DCI. Sex, age, H-H grade, mFS grade, preoperative mRS score, smoking, drinking, and hypertension were not associated with any increased risk of DCI in the analysis (*P* > 0.05). The relationship between age, H-H grade, ICPmax value, and DCI were present in Figure 4.

**TABLE 2:** Logistic regression analysis for risk factors of DCI.

	Univariable		Multivariable	
		
Covariate	OR (95% CI)	*P* value	OR (95% CI)	*P* value
Sex	1.597 (0.601–4.248)	0.348	1.381 (0.380–5.022)	0.624
Age	0.986 (0.941–1.033)	0.551	0.999 (0.937–1.064)	0.964
H-H grade	2.138 (1.025–4.459)	0.043	1.089 (0.191–6.214)	0.924
mFS score	1.390 (0.751–2.574)	0.294	1.301 (0.597–2.834)	0.507
mRS score	1.534 (1.033–2.276	0.034	1.327 (0.521–3.381)	0.553
Smoking	2.643 (0.669–10.440)	0.166	1.378 (0.097–19.648)	0.813
Drinking	3.391 (0.575–19.987)	0.177	3.271 (0.140–76.202)	0.461
Hypertension	0.655 (0.245–1.748)	0.398	0.373 (0.108–1.287)	0.118
ICPmax > 28.5 mmHg	4.442 (1.509–13.082)	0.007	4.351 (1.140–16.608)	0.031

*H-H grade, Hunt-Hess grade; mRS score, modified Rankin Scale score; mFS grade, modified Fisher grade; ICP, intracranial pressure.*

### The Relationship Between Intracranial Pressure Monitoring Guided Treatment and Outcomes

To evaluate whether aSAH patients could benefit from the ICP-directed treatment. We compare our cohort’s postoperative complications and neurological functions with patients treated at another ward (non-ICP monitoring) of our hospital at the same period. Baseline characteristics differed concerning H-H grade and aneurysm location (Table 3). Propensity score matching was conducted to make the comparison cohorts more similar; we matched 26 patients with ICP monitoring to 26 patients without ICP monitoring. No significant differences were found in sex, age, location of the ruptured aneurysm, H-H grade, mFS grade, current smoking, alcohol use, history of hypertension, hyperlipidemia, diabetes mellitus. The outcomes between patients with and without ICP monitoring in match groups were presented in Table 4. Patients with ICP-directed treatment showed a significantly lower incidence of DCI compared to patients without ICP monitoring.

**TABLE 3:** Baseline characteristics in patients with and without ICP monitoring.

Characteristic	Before PSM			After PSM		
		
	No-ICP (76)	ICP (68)	*P* value	No-ICP (26)	ICP (26)	*P* value
Age, years	52.9 ± 11.2	53.5 ± 10.5	0.742	51.7 ± 11.3	51.7 ± 10.2	0.975
Sex			0.210			0.768
Male	29 (38.2)	33 (48.5)		9 (34.6)	8 (30.8)	
Female	47 (61.8)	35 (51.5)		17 (65.4)	18 (69.2)	
H-H grade			<0.001			0.477
1	36 (47.4)	3 (4.4)		3 (11.6)	3 (11.6)	
2	8 (10.5)	49 (72.1)		7 (26.9)	11 (42.2)	
3	24 (31.6)	9 (13.2)		9 (34.6)	9 (34.6)	
4	8 (10.5)	7 (10.3)		7 (26.9)	3 (11.6)	
mFS score			0.100			0.170
2	25 (32.9)	20 (29.4)		5 (19.2)	4 (15.4)	
3	15 (19.7)	24 (35.3)		4 (15.4)	10 (38.5)	
4	36 (47.4)	24 (35.3)		17 (65.4)	12 (46.1)	
mRS score			0.445			0.239
1	53 (69.7)	54 (79.4)		12 (46.1)	17 (65.4)	
2	5 (6.6)	1 (1.5)		4 (15.4)	0 (0)	
3	4 (5.3)	3 (4.4)		2 (7.7)	2 (7.7)	
4	8 (10.5)	4 (5.9)		3 (11.6)	4 (15.4)	
5	6 (7.9)	6 (8.8)		5 (19.2)	3 (11.6)	
Location			0.027			0.566
ICA	3 (3.9)	5 (7.4)		3 (11.6)	1 (3.8)	
MCA	19 (25.0)	29 (42.7)		5 (19.2)	10 (38.5)	
ACoA	26 (34.2)	24 (35.3)		10 (38.5)	8 (30.7)	
PCoA	19 (25.0)	9 (13.1)		6 (23.1)	6 (23.1)	
ACA	4 (5.3)	1 (1.5)		1 (3.8)	1 (3.8)	
Posterior circulation	5 (6.6)	0 (0)		1 (3.8)	0 (0)	
Smoking	20 (26.3)	10 (14.7)	0.087	5 (19.2)	4 (15.4)	0.714
Drinking	13 (17.1)	6 (8.8)	0.143	4 (15.4)	2 (7.7)	0.385
Hypertension	48 (63.2)	32 (47.1)	0.064	15 (57.7)	12 (46.1)	0.405
Diabetes	6 (7.9)	1 (1.5)	0.074	3 (11.6)	0 (0)	0.074
Hyperlipidemia	1 (1.3)	1 (1.5)	0.937	1 (3.8)	1 (3.8)	1.000

*H-H grade, Hunt-Hess grade; mFS grade, modified Fisher grade; mRS score, modified Rankin Scale score; ICA, internal carotid artery; MCA, middle cerebral artery; ACoA, anterior communicating artery; PCoA, posterior communicating artery; ACA, anterior cerebral artery; ICP, intracranial pressure. Data are n (%) unless otherwise indicated. Mean values are given with SDs.*

**TABLE 4:** Outcome between patients with and without ICP monitoring in match groups.

	No-ICP monitoring	ICP monitoring	*P* value
Characteristic	*N* = 26	*N* = 26	
Post-op complications; F# (%):M# (%)	8 (30.8, 4:4)	2 (7.7, 1:1)	0.035
Ischemic stroke; F# (%):M# (%)	5 (19.2, 3:2)	1 (3.8, 1:0)	0.083
DCI; F# (%):M# (%)	3 (11.6, 1:2)	1 (3.8, 1:0)	0.012
Hydrocephalus; F# (%):M# (%)	2 (7.7, 1:1)	1 (3.8, 0:1)	0.552
Infection; F# (%):M# (%)	3 (11.6, 3:0)	4 (15.4, 1:3)	0.643
Survival; F# (%):M# (%)	23 (88.5, 17:6)	26 (100.0, 18:8)	0.074
Dependency; F# (%):M# (%)	13 (50.0, 11:2)	15 (57.7, 12:3)	0.578
Good prognosis; F# (%):M# (%)	9 (34.6, 7:2)	13 (50.0, 10:3)	0.262

*DCI, delayed cerebral ischemia. Data are n (%) unless otherwise indicated.*

## Discussion

Although the ICP elevation is a well-recognized phenomenon in aSAH that has been demonstrated to lead to poor outcomes, the value of ICP monitoring remains elusive. The precise thresholds of when ICP and blood flow begin to harm the brain are not known with certainty. Studies to date have tended to report ICP elevation according to the guideline for managing traumatic brain injury. The thresholds of ICP with the strongest statistical association with the outcome may not be suitable for aSAH patients. Thus, our analysis tended to provide new insights into the association between ICP and DCI following aSAH. Also, the value of ICP monitoring in aSAH patients was preliminarily explored in the present study.

There is no doubt that normal ICP is difficult to define (Czosnyka and Pickard, 2004), and previous studies have also reported numerous normal ranges (Hawryluk et al., 2020a). Considering it’s unethical to monitor ICP in healthy patients. Thus, the reported normal ICP is neither definite nor accurate. Complicating matters, a “normal” ICP has been reported to vary age and body position (Czosnyka and Pickard, 2004; Hawryluk et al., 2020a,b). In the present study, a discrete mode of 14 mmHg was consistently seen across distinct epochs for all patients (Figure 1). It is not suitable to infer normal ICP values from patients being treated for aSAH. Nevertheless, these data may somewhat indicate that less than 15 mmHg reflects normal ICP values, at least in those undergoing monitoring in the ICU. This result is also consistent with which reported in the published study (Marshall et al., 1979).

For patients with aSAH, the subarachnoid space and ventricle are full of hemoglobin. After the erythrocytic membranes become unstable and lyse, the hemoglobin releases oxyhemoglobin and other vasoactive blood products. The toxicity of oxyhemoglobin and other vasoactive blood products is the ultimate source of vascular dysfunction leading to micro thrombosis and vasospasm (Dodd et al., 2021). Further, microthrombi formed in the perivascular spaces after SAH can obstruct cerebrospinal fluid flow through the lymphatic system and contribute to increased ICP (Siler et al., 2014). As the ICP elevated gradually approaches mean arterial pressure, global ischemia developed, contributing to the DCI (Hayashi et al., 2000). In the present study, we also observed that the ICPmax value in patients with DCI was significantly higher than patients without DCI. Also, the ICP values of patients are most likely to be elevated on the 4th day after surgery and have the peak value at about the 5th day (Figure 2A). The deterioration of the clinical manifestation attributed to the DCI also occurred around 4–10 days post-SAH (Roos et al., 2000; Topkoru et al., 2017), which is highly consistent with our results. Our data also presented when ICP is around 30 mmHg, the difference between the proportion of patients with DCI and those without DCI is the largest (Figure 2B). It may indicate that the risk of DCI increases sharply at the point that ICP values approximately 30 mmHg. Using ROC analysis, we obtained the best ICP threshold for predicting DCI was 28.5 mmHg (Figure 3). After adjusting for other confounding factors, the ICPmax value >28.5 mmHg still showed a significant association with DCI occurrence. It is also consistent with evidence for a higher ICP threshold for mortality than for good outcomes (Sorrentino et al., 2012).

The use of hyperosmolar agents such as mannitol and hypertonic saline to control ICP after microsurgical clipping in aSAH patients is still controversial (Lee et al., 2006). Our institution is one of the largest neurosurgery centers in our country. Four professional cerebrovascular wards can provide treatment for aSAH patients. Thus, to investigate whether ICP-directed treatment improves the outcome of aSAH patients, the cohort that received ICP-directed treatment in the present study was compared to patients who were contemporaneously treated at the other wards. Although the comparison cohort taken from another ward was not set up to be the comparison cohort before the data was collected, these patients all underwent microsurgical clipping, and the postoperative medical treatment aside from the ICP monitoring and associated therapies didn’t differ between the two wards, which somewhat reduced selection bias. Further, we used PSM to adjust for potential baseline confounding characteristics between the two groups. Our data demonstrated that the incidence of DCI was significantly lower in the ICP monitoring group than the non-ICP monitoring groups, as well as the total postoperative complications. There was no significant difference in the neurological status at discharge between the two groups, which may attribute to the small sample size in match pairs. Although long-term follow-up can better evaluate the prognosis of patients. Considering the high rate of loss to follow-up the discharge neurological status was necessary.

## Limitations

Our study has several important limitations that need to be addressed for the accurate interpretation of our data. First, the results of this study only pertain to patients who are judged appropriate for ICP monitoring. The present study describes patients from a single institution. As the ICP values were recorded every 1-h epochs, the ICP-directed treatment in our cohort is not initiated until the 20 mmHg threshold has been exceeded for 1 h. It may investigate the pathological process better with higher-frequency data. Also, the thresholds that we identified may be confounded by ICP-directed treatments used to maintain ICP less than 20 mmHg and toxicities of these treatments. Moreover, we used the mRS score to evaluate the prognosis of all patients, while other studies used the Glasgow Outcome Scale. The use of discharge neurological status was suboptimal but necessary because of a high rate of loss to follow-up.

## Conclusion

Our study suggests that ICPs less than 15 mmHg possibly constitute normal values and that 28.5 mmHg is the ICP threshold most strongly associated with the occurrence of DCI in aSAH patients. Patients who received the ICP-directed treatment presented a lower incidence of DCI. Our findings provide a basis for the recommendation of ICP-directed treatment after aSAH.

## Data Availability Statement

The original contributions presented in the study are included in the article/Supplementary Material, further inquiries can be directed to the corresponding author. All results of the present study will be published in peer-reviewed journals and presented at relevant conferences.

## Ethics Statement

The studies involving human participants were reviewed and approved by IRB of Beijing Tiantan Hospital, Capital Medical University. The protocol for the LongTEAM study was approved by the Ethics Committee of Beijing Tiantan Hospital, Capital Medical University (KY 2021-008-01). Written informed consent to participate in this study was provided by the participants’ legal guardian/next of kin.

## Author Contributions

XC: conception and design, reviewing submitted version of manuscript, and study supervision. JY, JL, RnL, FL, YC, HH, DY, RiL, ZL, HZ, KY, HL, LZ, GS, and JZ: acquisition of data. JY: drafting the article. JY and JL: statistical analysis. SW, YZ, and XC: administrative, technical, and material support. All authors analysis and interpretation of data.

## Conflict of Interest

The authors declare that the research was conducted in the absence of any commercial or financial relationships that could be construed as a potential conflict of interest.

## Publisher’s Note

All claims expressed in this article are solely those of the authors and do not necessarily represent those of their affiliated organizations, or those of the publisher, the editors and the reviewers. Any product that may be evaluated in this article, or claim that may be made by its manufacturer, is not guaranteed or endorsed by the publisher.
